# EPHB4 Regulates the Proliferation and Metastasis of Oral Squamous Cell Carcinoma through the HMGB1/NF-κB Signalling Pathway

**DOI:** 10.7150/jca.59331

**Published:** 2021-08-20

**Authors:** Chen Yi, Xiliu Zhang, Hongyu Li, Guanhui Chen, Binghui Zeng, Yiming Li, Chao Wang, Yi He, Xun Chen, Zixian Huang, Dongsheng Yu

**Affiliations:** 1 Department of Oral and Maxillofacial Surgery, Hospital of Stomatology, Guanghua School of Stomatology, Sun Yat-sen University. Guangzhou, Guangdong, China, 510055.; 2 Guangdong Provincial Key Laboratory of Stomatology. Guangzhou, Guangdong, China, 510055.; 3 Department of Stomatology, the Seventh Affiliated Hospital, Sun Yat-sen University, Shenzhen. Guangdong, China, 518107.; 4 Department of Oral and Maxillofacial Surgery, Sun Yat-sen Memorial Hospital, Sun Yat-sen University, Guangzhou, Guangdong, China, 510120.

**Keywords:** Oral squamous cell carcinoma, proliferation, cervical lymphatic metastasis, EPHB4, HMGB1, NF-κB

## Abstract

**Background:** Malignant proliferation and cervical lymphatic metastasis restrict the prognosis of oral squamous cell carcinoma (OSCC). Erythropoietin-producing human hepatocellular B4 (EPHB4) regulates a series of tumour functions involving tumourigenesis, cancer cell attachment and metastasis. However, the mechanism of EphB4 regulating the malignant progression of OSCC has not been fully elucidated.

**Methods:** EPHB4 expression was analysed in 65 OSCC samples and adjacent noncancerous tissues through immunohistochemistry (IHC). siRNA and overexpression plasmids were transfected into OSCC cells to modify EPHB4 expression, and then, regulatory functions were explored *in vitro* and *in vivo*. Co-immunoprecipitation (Co-IP) and mass spectrometry were applied to detect proteins interacting with EPHB4. Subsequently, protein stability assays and NF-κB pathway inhibition assays were used to verify the regulation of EPHB4, high-mobility group box 1 (HMGB1) and nuclear factor-κB (NF-κB) activation.

**Results:** EPHB4 was found to be highly expressed in OSCC tissues, which was related to tumour stage and lymphatic metastasis and resulted in a poor prognosis. Cellular experiments and mouse tongue xenograft models further confirmed that high EPHB4 expression promoted the proliferation and metastasis of OSCC tumours. Mechanistically, co-IP and mass spectrometry studies indicated that EPHB4 could bind to HMGB1 and maintain HMGB1 stability. Downregulation of HMGB1 inhibited the proliferation and metastasis of OSCC cells and inhibited NF-κB phosphorylation activation but did not affect EPHB4 expression.

**Conclusion:** This study revealed the mechanism by which EPHB4 promotes the proliferation and metastasis of OSCC by activating the HMGB1-mediated NF-κB signalling pathway, which can be exploited as a novel marker or therapeutic target to control metastasis and improve the survival rate of OSCC.

## Introduction

Oral squamous cell carcinoma (OSCC) is one of the most aggressive malignant tumours worldwide 1. There were nearly 377,713 new cases of lip and oral cancer in 2020, accounting for nearly 2% of all sites. Moreover, approximately 177,757 lives are lost each year due to OSCC progression and recurrence 2. Malignant proliferation and lymph node metastasis have been proven to be two significant factors limiting the survival rate of OSCC patients 3 and have received substantial attention in the field of cancer research 4. At present, many genes and signal transduction pathways regulating tumourigenesis and metastasis have been discovered 5. However, the specific mechanisms related to OSCC tumour proliferation and metastasis have not been fully elucidated 3, 6. Therefore, there is a pressing need to search for more reliable biomarkers that can become the key targets of OSCC treatment by inhibiting proliferation and metastasis.

The erythropoietin-producing human hepatocellular (EPH) receptor family, as the largest subfamily of receptor tyrosine kinases (RTKs), has been demonstrated to play widespread roles during physiological and pathological processes 7. For instance, EPHA2 was proven to be abnormally expressed in prostate, lung, and oesophageal cancers 8, and EPHB6 promotes both malignant progression and chemosensitivity in breast cancer 9. As a typical member of the EPH family, EPHB4 can regulate a series of tumour cell functions involving tumourigenesis, cancer cell attachment, angiogenesis, migration and invasion through bidirectional signals 10. Moreover, research on EPHB4-targeted drugs is progressing, and corresponding specific inhibitors have been evaluated in clinical trials 11. However, the expression and function of EPHB4 in OSCC are still unclear, and further detailed studies of its involvement in OSCC progression are necessary.

In our preliminary study, EPHB4 could regulate the proliferative and metastatic ability of OSCC, and co-IP and mass spectrometry revealed that it could interact with high-mobility group box 1 (HMGB1), which was reported to participate in cellular physiological activities such as cell proliferation, metastasis, and DNA repair 12, 13. Furthermore, HMGB1 might regulate the activation of nuclear factor-κB (NF-κB) 14. Hence, the data indicate that EPHB4 expression regulates OSCC cell function by regulating HMGB1-mediated NF-κB pathways, which eventually promote OSCC malignant progression.

## Materials and methods

### Reagents and antibodies

The culture medium (DMEM, DMEM-F12) and foetal bovine serum (FBS) were purchased from Gibco (NY, USA). Antibodies against EPHB4 (#14960), P-P65 (#3033), P65 (#8242), P-IκBα (#2859), IκBα (#4814), and GAPDH (#5174) were purchased from Cell Signaling Technology (Beverly, MA, USA). Antibodies against HMGB1 (#ab18256) were purchased from Abcam (UK). Secondary antibodies involving goat anti-rabbit and anti-mouse IgG-HRP were purchased from Santa Cruz (USA). The NF-κB inhibitor was purchased from MCE (JSH-23, #HY-13982, NJ, USA), and cycloheximide (CHX) was obtained from ApexBio (#A8244, Houston, TX, USA). Other chemical products were purchased from Sigma (St. Louis, MO, USA), or Thermo Fisher Scientific (Waltham, MA, USA).

### Patients and clinical samples

A total of 65 OSCC tissues and matched adjacent noncancerous tissue paraffin sections were collected from patients who underwent surgery between June 2014 and December 2015 at the Hospital of Stomatology, Sun Yat-Sen University. The inclusion criteria included the following: I. a pathological diagnosis of OSCC; II. patient must be willing to participate in the subsequent follow-up; and III. the type of tumour must be primary cancer. The exclusion criteria were as follows: I. the receipt of chemotherapy or radiotherapy; II. a diagnosis of multiple cancers; and III. the presence of serious cardiovascular and cerebrovascular diseases. Apart from this information, age, sex, clinical stage, tumour differentiation status and lymphatic metastasis status of the OSCC patients were collected. This study was approved by the ethics committee of the Hospital of Stomatology of Sun Yat-Sen University. Informed consent was acquired from all patients.

### Immunohistochemical (IHC) analysis

After deparaffinization of the paraffin-embedded tissue sections, antigen retrieval was performed by boiling the tissue slides in a pressure cooker with sodium citrate buffer (10 mM, pH 6.0). The slides were then incubated overnight at 4 °C with the indicated primary antibodies after blocking with 3% H_2_O_2_ and normal goat serum. Then, we used PBST (PSB + 1% Tween) to rinse the samples three times and incubated the tissues with the corresponding secondary antibody at room temperature for 30 minutes. Subsequently, the slides were stained with 3-3′-diaminobenzidine, counterstained with haematoxylin and mounted under a coverslip. The images were observed under a Zeiss microscope. Histochemistry total scores were equal to the product of positive cell rate score [scored as 0 (0%), 1 (1-25%), 2 (26-50%) and 3 (>50%)] multiplied by the score of staining intensity (ranging from 0 to 3), which was evaluated by 2 pathologists. Scores above 3 were considered high expression, and scores below 3 were classified as low expression.

### Cell culture

The SCC9 cell line was obtained from American Type Culture Collection (ATCC, USA). Normal oral keratinocytes (NOK) and UM1 cell lines were purchased from the Cell Bank of the Chinese Academy of Sciences (Shanghai, China). OSCC cells (SCC9 and UM1) were routinely cultured in DMEM-F12 supplemented with 10% FBS (Invitrogen, USA), while the NOK cells were cultured in defined keratinocyte growth medium (Gibco, USA). The cells mentioned above were cultured in a 37 °C humidified incubator containing 5% CO_2_. All cell lines were confirmed by short tandem repeat profiling analysis and were free of mycoplasma contamination.

### Cell transfection

Transient transfection sequences consisted of several groups of small interfering RNAs (siRNAs), including control siRNA (siNC) and four other siRNAs targeting EPHB4 and HMGB1 separately (siB4-1, siB4-2, siHM-1, and siHM-2), which were synthesized by IGEbio (Guangzhou, China). Lipofectamine 3000 (Invitrogen, USA) was used for transient transfection following reagent specification. The siRNA sequences are shown as follows:

For stable transfection, the EPHB4 or luciferase gene was first constructed into the pCDH or PLKO plasmid; then, the plasmid was co-transfected with pMD2.G and pSPAX2 at a proportion of 5:2:3 into 293T cells together. After 48 hours of transfection, the supernatants were collected and applied to infected OSCC cells. Forty-eight hours later, puromycin (50 mg/ml, 1:2000) was added to the culture medium to select for stable-expression cells. Western blotting and PCR analyses were performed for further confirmation.

### Quantitative real-time PCR

Total RNA was extracted from cells with RNAzol (#RN 190, Invitrogen, Carlsbad, CA, USA) following the manufacturer's instructions. The extracted RNA was reverse transcribed into cDNA with Prime-Script™ RT Master Mix (TaKaRa, Japan) on an ABI 9700 Real-Time PCR system (ABI, USA), and cDNA amplification was subsequently conducted using the Roche Light Cycler 480 Real-Time System (Roche, Germany) with TB Green Premix Ex (TaKaRa, Japan). The primers for EPHB4 and HMGB1 were purchased from Ruibiotech (Beijing, China). The primer sequences are as follows:

qRT-PCR was performed using the primers described above, and the reaction conditions were as follows: part I, predenaturation involved a reaction of 95 °C for 30 s; part II, PCR parameters were 95 °C for 5 s, 55 °C for 30 s and 72 °C for 30 s for 40 cycles; and part III, melting involved 95 °C for 5 s, 60 °C for 60 s and 95°C for 1 s for 1 cycle. The final stage ended with cooling to 50 °C for 30 s. The relative expression of target mRNA was detected simultaneously using the Roche Light Cycler 480 Real-time PCR instrument (Roche, USA). Every experiment was repeated three times.

### Western blot analysis

Total cell proteins were extracted using lysis buffer containing proteinase inhibitor and phosphatase inhibitor (Beyotime, Beijing, China) following the instructions. After the protein was quantified using the BCA kit (CWBio, Beijing, China), equal amounts of denatured protein samples were electrophoretically separated by SDS-PAGE gels and transferred to polyvinylidene fluoride (PVDF) membranes (IPVH00010; Millipore, USA). After blocking with 5% nonfat milk, the blots were incubated with the indicated primary antibodies at 4 °C overnight. The next day, blots were washed in TBST 3 times and then incubated with the appropriate secondary antibodies for 1 hour at RT. Detection was performed according to standard protocols.

### Wound-healing and transwell assays

For the wound-healing assay, treated cells were plated into 6-well culture plates, and a cell-free growth zone was marked out when the cells covered 90% of the plate. Then, cell images were captured at 0, 12, 24, 48 and 72 hours after scratching.

Transwell chambers were used for cell migration and invasion assays according to the manufacturer's specifications. After treated cells were trypsinized and resuspended in serum-free DMEM/F12 medium, 50,000 cells per 100 µl of medium per well were placed onto the upper chambers (Falcon) with an 8-μm pore membrane, while 700 µl of the same culture medium with 10% FBS was added to the lower chambers serving as a chemoattractant. After a 24- to 48-hour period of cell-directed motion, the cells remaining on the upper surface of the membrane were gently wiped away with a cotton swab, while those migrating to the bottom membrane were fixed, dyed with crystal violet, and counted in five random fields under a 10× objective lens of a light microscope (Zeiss). For the cell invasion assay, 100,000 cells in 100 µl of medium per well were placed onto the upper chambers. Except for the upper chambers precoated with Matrigel (BD Biosciences, NJ, USA) diluted 10 times in serum-free medium, the rest of this assay was similar to the migration assay described above. These experiments were all repeated three times. The number of cells was counted by three researchers separately.

### Cell proliferation and colony-formation assay

Twenty-four hours after transfection, 2000 cells/well were seeded in 96-well plates and incubated overnight in complete medium. Cell Counting Kit (CCK)-8 (Dojindo Laboratories, Tokyo, Japan) assays were used to evaluate cell viability at 24, 48, 72, 96, and 108 hours according to the manufacturer's specifications. After CCK8 reagent was added to each well for a 2-hour incubation, the absorbance was measured at 450 nm using a microplate spectrophotometer (Bio-Rad, Winooski, VT, USA). The resulting data were recorded as the original OD values, and every experiment was repeated three times.

In the colony-formation assay, 600 treated cells/well were seeded in 6-well culture plates. After a 10-12-day incubation period, the cells were fixed with paraformaldehyde for 20 minutes and subsequently stained with crystal violet for 10 minutes. A cell mass of more than 50 cells was counted by microscopy. Control groups were included for each experiment, and every experiment was repeated three times.

### Immunoprecipitation (IP) assay and mass spectrometry analysis

Total cell protein was extracted using NP-40 lysis buffer (Beyotime, China) and first incubated with primary antibodies (anti-EPHB4 or anti-HMGB1) or IgG (as control) at 4 °C for 2 hours and subsequently fully mixed with protein A/G agarose (Santa Cruz, Texas, USA) overnight at 4 °C according to the manufacturer's specifications. Beads holding the protein-antibody complex were washed with NP-40 3 times, and then washed bead-protein-antibody complexes were denatured with 5× SDS loading buffer at 100 °C for 10 minutes. After centrifugation at 13,000 rpm for 10 minutes, the supernatants were collected for subsequent western blot analysis to identify protein-protein interactions.

For mass spectrometry, protein immunoprecipitated by anti-EPHB4 antibody was digested, and proteomics screening was performed by mass spectrometry analysis.

### *In vivo* tumour xenograft model and cervical metastasis

All animal experiments described in this article were approved by the Sun Yat-sen University Institutional Animal Care and Use Committee and performed following the National Institutes of Health Guide for the Care and Use of Laboratory Animals.

Four- to five-week-old female BALB/c nude mice were supplied by the Animal Feeding Center of Sun Yat-sen University and were placed in an SPF barrier environment at the Department of Laboratory Animals of Sun Yat-sen University.

SCC9 cell lines containing the luciferase gene were used for *in vivo* tumourigenesis and metastasis assays. A total of 20 nude mice were randomly divided into 4 groups. Then, four groups of stable cells (5×10^5^), including the EPHB4-overexpression group (SCC9-EPHB4) and its control group (control-1) and the EPHB4-knockdown group (shEPHB4) and its control group (control-2), were injected into the left lingual flange of each mouse. Tumour formation was monitored over a 3-week period, and the tumour volume was calculated as V = length × width^2^ × 0.5. When the average tumour volume reached 5 × 5 mm (length × width, 60 mm^3^), D-luciferin (#12511, AAT Bioquest) was injected intraperitoneally 10-15 minutes before imaging at 150 mg/kg to observe metastasis of the tumour using *In vivo* Optical Imaging Technology (PerkinElmer). Then, the mice were sacrificed, and tumours and metastatic lymph nodes were collected and paraffin-embedded for IHC.

### Statistical analysis

All statistical analyses in this article were performed using GraphPad Prism 7 and SPSS version 20.0. An unpaired Student's t-test was used for two-group quantitative comparisons, such as the comparison of EPHB4 expression in OSCC tissues and adjacent noncancerous (ANC) tissues. Two-way ANOVA was used for multiple group comparisons. The correlation between EPHB4 expression and patients' clinicopathological characteristics was evaluated by Kruskal-Wallis analysis. Moreover, Kaplan-Meier analysis and log-rank tests were applied for patient survival analysis. Student's t-test was also used to compare the PCR, cell proliferation, migration and invasion, and tumour xenograft results. Unless otherwise noted, quantitative data are expressed as the mean and standard error of the mean (SD). All experiments were repeated 2-3 times, and a *p*-value of *p* < 0.05 (*p< 0.01; **p < 0.001) was considered significant.

## Results

### EPHB4 was highly expressed in OSCC and associated with clinical stage, lymph node metastasis and a poor prognosis

**Figure 1:**
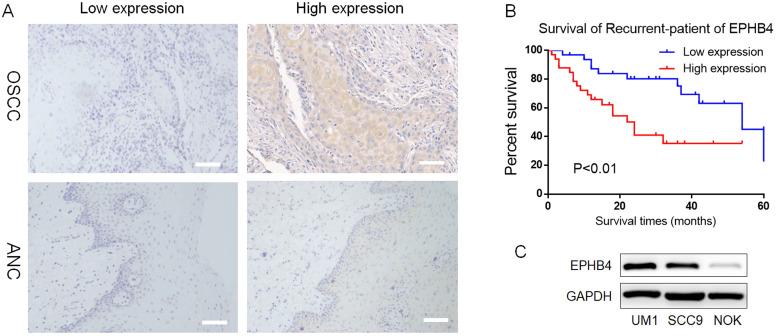
** EPHB4 is highly expressed in OSCC compared with ANC tissues. A.** Typical IHC results representing low and high EPHB4 expression in OSCC and ANC samples are shown (scale bar, 100 μm). **B.** Kaplan-Meier analysis revealed that patients with higher EPHB4 expression had a lower survival rate and worse prognosis. **C.** Western blot analysis confirmed that EPHB4 expression in OSCC cell lines (UM1 and SCC9) was significantly higher than that in NOK cells.

To investigate the clinical significance of EPHB4 expression in OSCC, 65 OSCC tissues and their adjacent noncancerous (ANC) tissues were collected for immunohistochemical staining (IHC). The high EPHB4 expression rate in OSCC samples was 33/65 (50.77%), which was notably higher than the 20/65 (30.77%) in ANC tissues (Table 1, Figure 1A). EPHB4 expression was not significantly related to the age or sex of OSCC patients (*p*>0.05, Table 2), but was positively correlated with lymph metastasis, clinical stage and differentiation (*p*<0.05, Table 2).

Furthermore, patients with higher EPHB4 expression had a lower survival rate and worse prognosis (*p*<0.01, Figure 1B). Therefore, EPHB4 may be a potential indicator of tumour metastasis and poor prognosis in OSCC patients, which indicates the great significance of studying the role of EPHB4 in OSCC.

Western blot results showed that EPHB4 expression in 2 OSCC cell lines, SCC9 and UM1, was higher than that in NOK (Figure 1C), which was consistent with previous IHC results.

### Downregulated EPHB4 expression inhibited the proliferative and metastatic ability of OSCC cells

**Figure 2:**
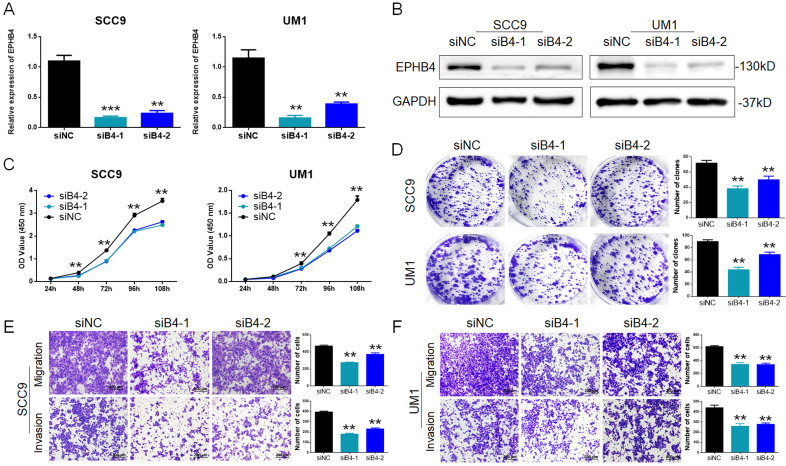
** Inhibition of EPHB4 reduced the proliferation, migration and invasion abilities of OSCC cells. A.** PCR detection revealed that EPHB4 expression was successfully inhibited in SCC9 and UM1 cells after siRNA transfection. **B.** Western blot analysis confirmed that EPHB4 was inhibited in SCC9 and UM1 cells after siRNA transfection. **C.** After inhibiting EPHB4, a CCK-8 assay showed that the proliferation ability of SCC9 cells and UM1 cells was significantly reduced. **D.** Downregulation of EPHB4 inhibited the colony-forming ability of SCC9 and UM1 cells. **E-F.** Transwell assays revealed that the number of SCC9 and UM1 cells migrating or invading the lower chamber was significantly reduced following EPHB4 downregulation, representing the decline of their migration and invasion ability (scale bar, 200 μm).

To study the specific regulation of EPHB4 in OSCC cells, siRNA (siB4) was applied to target and inhibit EPHB4 expression in SCC9 and UM1 cells. Then, the transfection efficiency of EPHB4 was confirmed to be successfully down-regulated by PCR and western blot (Figure 2A&B).

CCK-8 assays and colony-formation assays revealed that the downregulation of EPHB4 inhibited the proliferation of SCC9 and UM1 cells (Figure 2C&D). Transwell assays were applied to detect cell metastasis. After knocking down EPHB4, the migration and invasion ability of SCC9 and UM1 cells were significantly reduced (Figure 2E&F). In addition, wound-healing assays were performed and displayed the same trends (Figure 1sA&B).

### Upregulation of EPHB4 enhances OSCC cellular proliferative and metastatic ability

**Figure 3:**
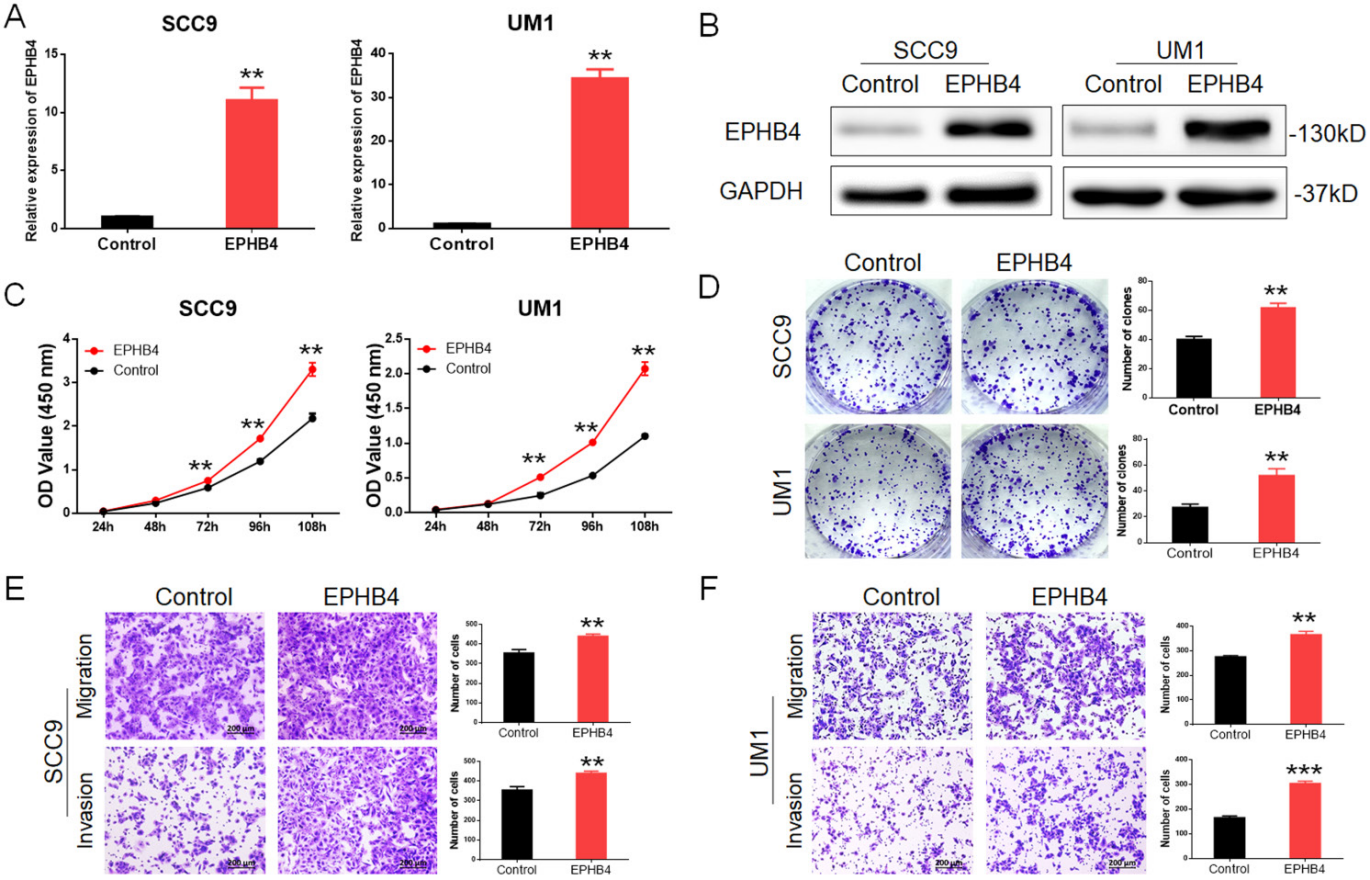
** Upregulation of EPHB4 enhances the proliferation, migration and invasion abilities of OSCC cells. A.** PCR revealed that EPHB4 expression was successfully up-regulated in SCC9 and UM1 cells after lentiviral transfection. **B.** Western blot analysis confirmed that EPHB4 was up-regulated in SCC9 and UM1 cells after lentiviral transfection. **C.** After upregulating the expression of EPHB4, the proliferative ability of SCC9 and UM1 cells was enhanced. **D.** Overexpression of EPHB4 enhanced the colony-forming ability of SCC9 and UM1 cells. **E-F.** Transwell assays demonstrated that the overexpression of EPHB4 promoted the migration and invasion ability of SCC9 and UM1 cells (scale bar, 200 μm).

We constructed EPHB4-overexpressing SCC9 and UM1 cells through lentiviral transfection, and the corresponding empty vectors were used as controls. EPHB4 expression was verified by protein and mRNA analysis (Figure 3A&B). After the overexpression of EPHB4, the proliferative and tumourigenesis abilities of SCC9 and UM1 cells were significantly enhanced (Figure 3C&D). Meanwhile, the results showed that the migration and invasion ability of the cells increased after upregulating EPHB4. The cells passing through the chamber semipermeable membrane increased significantly at the same time (Figure 3E&F), and the repair ability of the overexpression cell line was stronger after scratching (Figure 1 sC&D).

These results were consistent with those of previous knockdown experiments.

### EPHB4 regulated OSCC xenograft sizes and cervical lymphatic metastasis of tongue xenografts *in situ*

**Figure 4:**
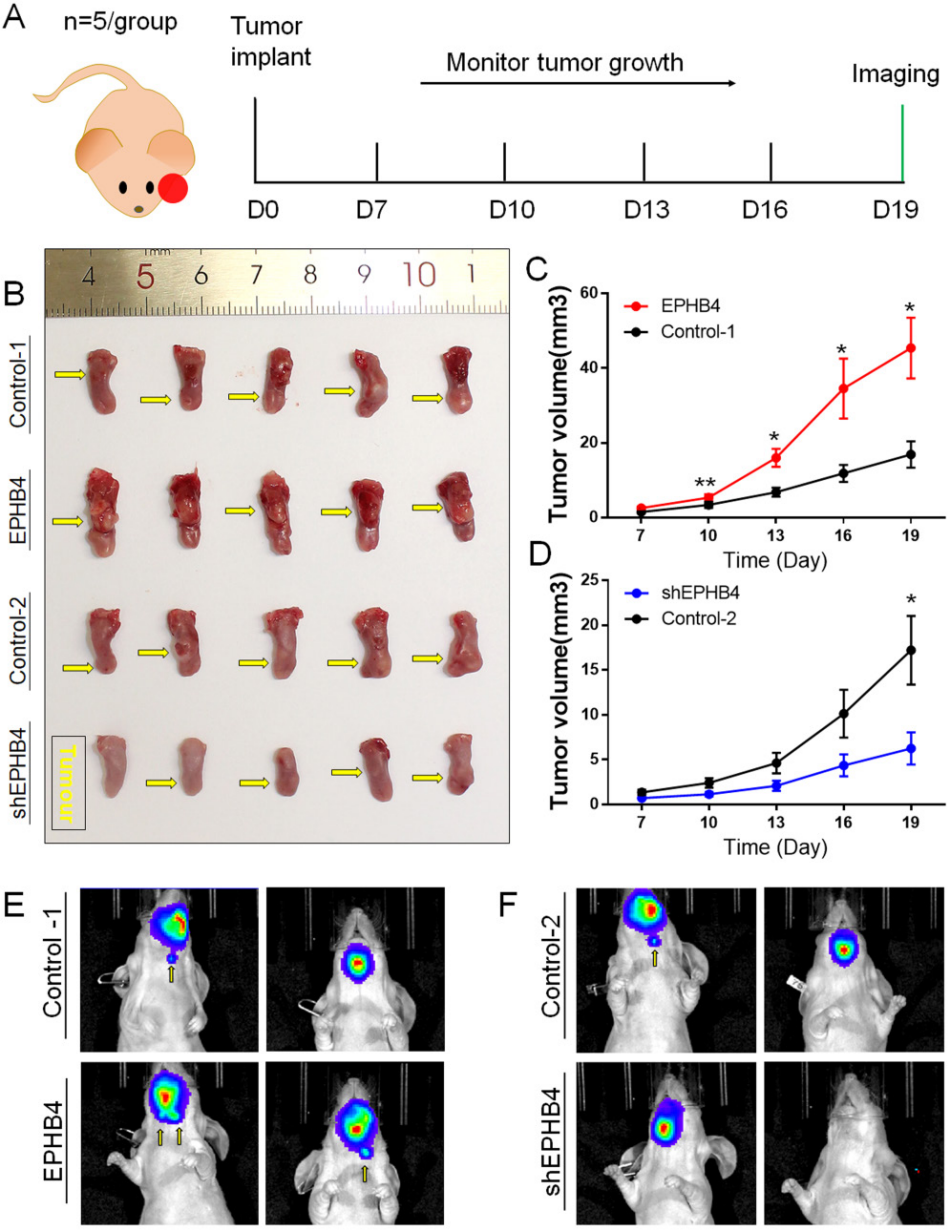
** EPHB4 promotes OSCC xenograft sizes and cervical lymphatic metastasis of tongue xenografts. A.** Schematic diagram of orthotopic xenografts of mouse tongue. **B.** On the 19^th^ day after tumour formation, the mouse tongues with tumours were obtained. The tumour volume of the EPHB4-overexpression group was larger than that of the control group. The tumour size of the shEPHB4 group was smaller than that of the control-2 group. The location of each tumour in the tongue is marked by a yellow arrow in the figure. **C-D.** The tumour growth curve shows that the growth rate was faster in the EPHB4-overexpression group than in the control group starting on the 10th day, while tumours in the shEPHB4 group grew slower than those in the control group. **E-F.** *In vivo* imaging experiments showed that cervical lymph node metastasis occurred earlier in the EPHB4-overexpression group than in the control group but less often in the EPHB4-knockdown group.

We generated four groups of xenograft *in situ* mouse models to evaluate the proliferative and metastatic abilities of EPHB4. Tumour formation was noted 7 days after SCC9 cell injection into the tongue of nude mice, and we performed tumour measurements every 3 days. Finally, on the 19th day after tumour formation, we performed live animal imaging observations using an *in vivo* imaging system. The schematic diagram is shown in Figure 4A.

Compared with the control-1 group, tumour growth was faster (Figure 4B&C), and cervical lymph node metastasis was more likely to occur (Figure 4E) in the EPHB4-overexpression group. The growth rate of the EPHB4-knockdown group was slower than that of the control-2 group (Figure 4B&D), and cervical lymph node metastasis was less frequent (Figure 4F).

In summary, EPHB4 was demonstrated to promote OSCC proliferation and metastasis by both *in vivo* and *in vitro* experiments.

### Mass spectrometry analysis revealed that EPHB4 interacted with HMGB1

Given the above results, it was clear that EPHB4 could regulate the proliferation, migration and invasion ability of OSCC. However, its regulatory mechanism is currently poorly studied. We applied mass spectrometry and biological analysis for further clarification.

**Figure 5:**
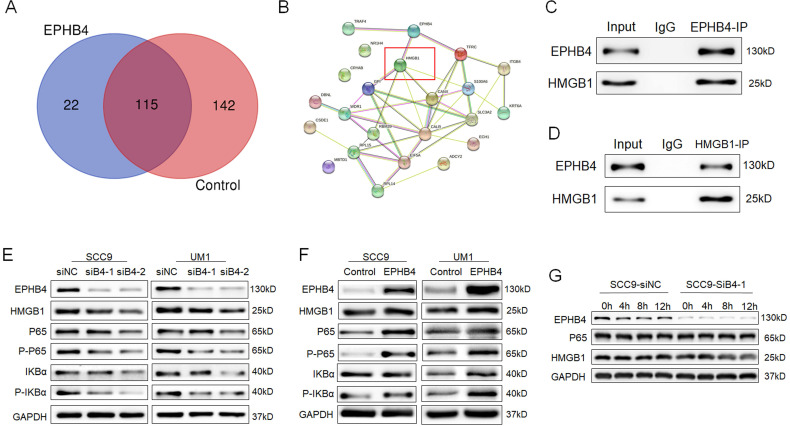
** EPHB4 interacted with HMGB1 and activated NF-κB signalling. A.** Mass spectrometry experiments detected 22 proteins that may specifically bind to EPHB4 in cells compared with control cells. **B.** HMGB1, which was identified to have a high binding efficiency to EHPB4, is marked with a red box. **C-D.** An endogenous EPHB4-IP assay verified that HMGB1 was immunoprecipitated by an anti-EPHB4 antibody, while an endogenous HMGB1-IP assay showed that EPHB4 could be immunoprecipitated by HMGB1 in turn, which jointly substantiated the mutual immunoprecipitation between EPHB4 and HMGB1. **E.** Western blot assays verified that when EPHB4 expression was down-regulated, HMGB1 expression decreased, and the expression of P-P65 and P-IKBα in the NF-κB signalling pathway was also inhibited. **F.** Conversely, P-P65 and P-IKBα were significantly increased in EPHB4-overexpressing cells. **G.** Protein stability experiments showed that HMGB1 stability decreased following the downregulation of EPHB4.

A total of 137 proteins that bind to EPHB4 in SCC9 cells were identified through mass spectrometry analysis (Figure 3s), and compared with the IgG control group, 22 of them specifically combined with EPHB4. The results of protein network analysis (https://string-db.org/) are shown in Figure 5A. EPHB4 and HMGB1 were identified to obtain relatively high binding efficiency and priority to the signals among the 22 candidate genes (Figure 5B, Table 3). IP analysis further demonstrated that EPHB4 coprecipitated with HMGB1 (Figure 5C&D). In addition, biological analysis revealed that HMGB1 is an important protein regulating NF-κB signalling. Hence, we continued to explore the potential interaction and mechanism between EPHB4 and HMGB1 in OSCC.

### EPHB4 regulated NF-κB signalling pathways via HMGB1

Mounting evidence shows that HMGB1 can promote the malignant progression of different types of cancer through the NF-κB signalling pathway 15-17. To explore whether HMGB1 plays a pivotal role in the regulation of EPHB4 and NF-κB signalling pathways in OSCC cells, western blot analysis was performed and showed that HMGB1 expression was down-regulated following EPHB4 suppression in SCC9 and UM1 cells and vice versa (Figure 5E, 5F). In addition, the phosphorylation of NF-κB pathway members P-P65 and P-IκBα was significantly down-regulated (Figure 5E). When EPHB4 expression was up-regulated, the phosphorylation of P65 and IκBα was increased (Figure 5F).

Additionally, we conducted a CHX assay to investigate the internal connection between EPHB4 and HMGB1 and found that the protein stability of HMGB1 was weakened following the downregulation of EPHB4 (Figure 5G). The above results indicated that the expression of HMGB1 and NF-κB pathway members changed following a change in EPHB4 expression.

### EPHB4 promoted OSCC cellular functions through the HMGB1-mediated NF-κB signalling pathway

**Figure 6:**
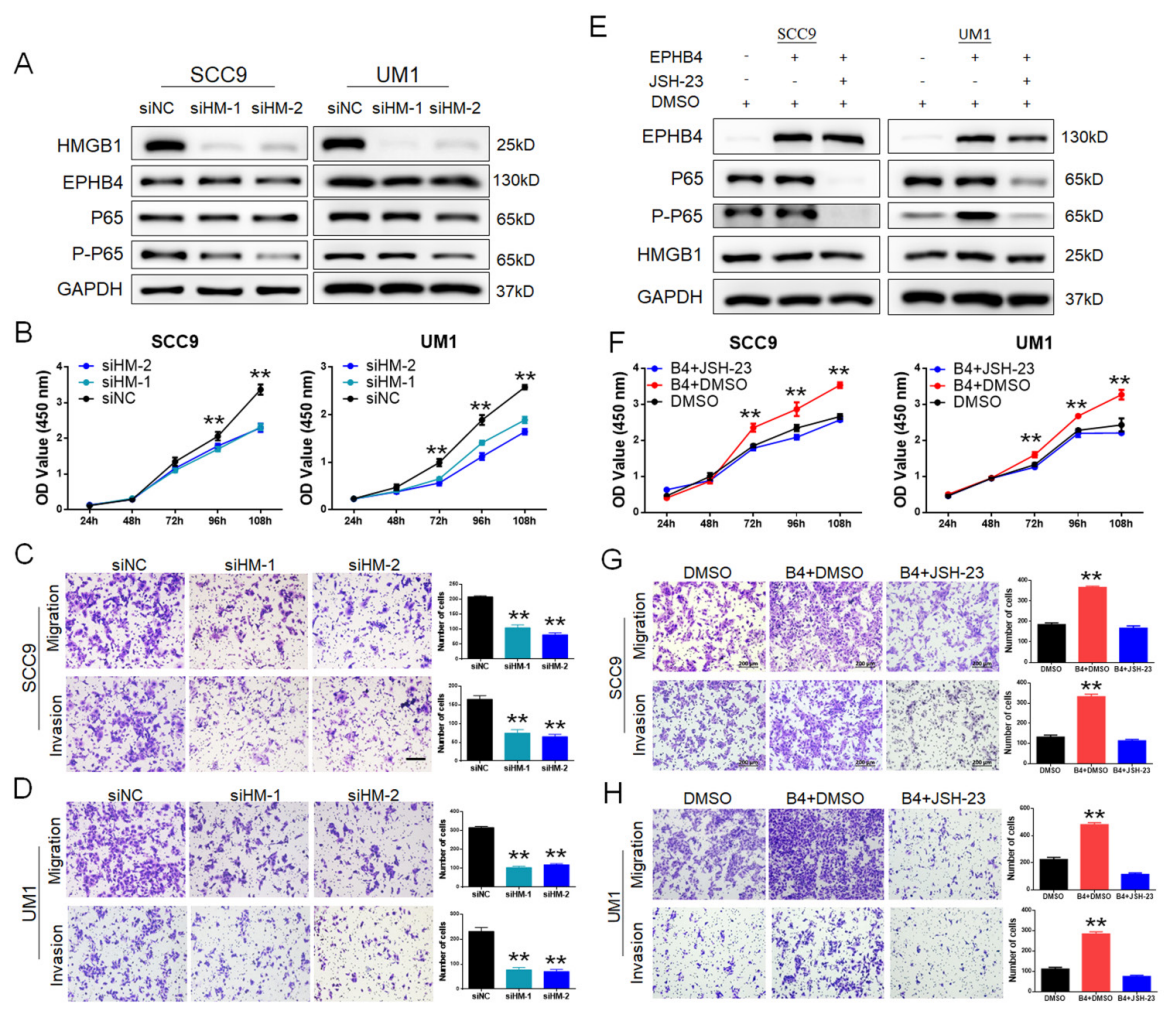
** EPHB4 regulates the functions of OSCC cells through the HMGB1-NF-κB signalling pathway. A.** EPHB4 expression did not change significantly following knockdown of HMGB1, while the expression of P-P65 decreased. **B.** After inhibiting HMGB1, the CCK-8 assay showed that the proliferation ability of SCC9 and UM1 cells was significantly reduced. **C-D.** Downregulation of HMGB1 dampened the migration and invasion ability of both SCC9 and UM1 cells. **E.** The NF-κB inhibitor JSH-23 was used to treat EPHB4-overexpressing cells, after which HMGB1 expression did not change significantly, while the expression of P-P65 was down-regulated. **F.** After the use of JSH-23, the proliferation ability of EPHB4-overexpressing SCC9 and UM1 cells was decreased. The proliferation-promoting effects of EPHB4 were rescued. **G-H.** The application of JSH-23 also suppressed EPHB4-overexpressing cell migration and invasion. The metastasis-activating effects of EPHB4 were blocked (scale bar, 200 μm).

To further clarify the regulatory mechanism of EPHB4-HMGB1, we designed siRNAs (siHM-1 and siHM-2) to knockdown HMGB1 expression in SCC9 and UM1 cells. The protein expression of EPHB4 did not change significantly, while the downstream factor P-P65 was down-regulated after inhibiting HMGB1 (Figure 6A). In addition, the proliferative ability (Figure 6B&4sA) and the migration and invasion ability (Figure 6C&D) of OSCC cells were significantly inhibited. Therefore, EPHB4 was shown to be the upstream regulator of HMGB1.

**Figure 7:**
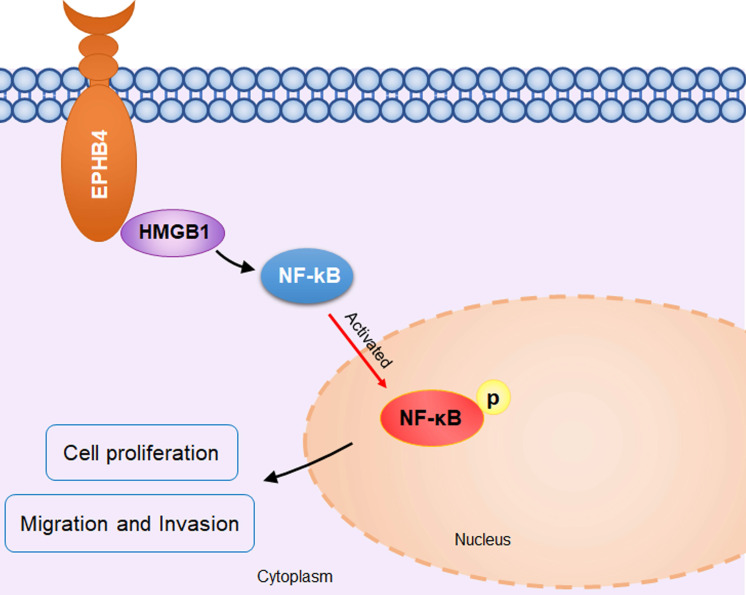
** Schematic diagram of EPHB4 promoting proliferation and metastasis via HMGB1-NF-κB in OSCC.** In OSCC cells, EPHB4 and HMGB1 bind to each other in the cytoplasm and activate downstream NF-kB through P65 phosphorylation, thereby promoting cellular proliferation, migration and invasion.

To confirm the regulatory relationship between EPHB4 and NF-κB, we used the inhibitor JSH-23 to inhibit NF-κB p65 in EPHB4-overexpressing SCC9 and UM1 cells. The results showed that P-P65 expression was significantly down-regulated with the use of JSH-23, while there was no significant effect on the expression of EPHB4 and HMGB1 (Figure 6E). With the application of the JSH-23 inhibitor, the proliferation, migration and invasion of SCC9 and UM1 cells were significantly reduced (Figure 6F, G, H, 4sB). On the basis of these results, EPHB4 may contribute to the malignant progression of OSCC through the HMGB1-mediated NF-κB signalling pathway, as shown in the mechanism diagram (Figure 7).

## Discussion

Malignant proliferation and cervical lymph node metastasis have become two major roadblocks that restrict the five-year survival rate of OSCC patients 18. At present, specific molecular OSCC prognosticators have only been partially identified 19. The limited success of molecular-targeted therapy for OSCC emphasizes the need to identify new treatment targets and biomarkers for OSCC 20.

In recent years, Eph receptors and their ligands have been shown to participate in maintaining cell homeostasis by regulating different functions of cells 7. In general, Eph receptors were demonstrated to generate cellular responses, including changes in cell motility and adhesion, in the actin cytoskeleton, as well as in the regulation of cell proliferation and survival 21-23. Moreover, accumulating evidence has indicated that the Eph/ephrin family is dysregulated in a variety of tumours, which can evoke bidirectional regulation of tumours through forward and reverse signaling 24. However, the mechanism of the regulation varies among cancer types 25, 26. As an important member of the EPH family, EPHB4 is usually overexpressed in several tumour types, such as ovarian 27, prostate 28, breast, oesophageal 29 and colon cancer 30. In contrast, EPHB4 was shown to be a tumour suppressor in intestinal carcinoma 31. Current studies have suggested that the mechanism by which EPHB4 plays a role in promoting or suppressing cancer remains elusive and is closely linked to its context in various types of tumours. Our data demonstrated the carcinogenic role of EphB4 in OSCC. We clarified that EPHB4 was highly expressed in OSCC compared with noncancerous tissues, which was consistent with the results of GEPIA (http://gepia.cancer-pku.cn/) database analysis. Furthermore, we found that EphB4 expression was related to OSCC differentiation, lymph node metastasis, clinical stage, and prognosis.

To date, the role of EPHB4 in HNSCC has rarely been described. Inhibition of EPHB4 was reported to enhance radiation sensitivity and chemosensitivity in HNSCC by promoting cell apoptosis and G2 cell cycle arrest 32, 33. Masood found that decreased EphB4 expression results in a significant inhibition of tumour growth in xenograft models 34. However, the downstream effector molecules by which EphB4 regulates the progression of OSCC and their interactions with other signalling pathways have not been fully elucidated. In our research, EPHB4 was demonstrated to enhance OSCC cell proliferation, migration, and invasion. Moreover, the tongue-transplanted tumours of nude mice showed that high EPHB4 expression promoted tumour growth and cervical lymph node metastasis. To clarify the internal molecular mechanism by which EphB4 regulates OSCC proliferation and metastasis, mass spectrometry was applied and revealed that EPHB4 could bind to HMGB1 and potentially affect NF-κB pathways.

As an evolutionarily conserved protein in most eukaryotes 35, the biological effects of HMGB1 depend on the state of the cell and its location in the cell 12. In the nucleus, HMGB1 participates in DNA repair, transcription, and telomere maintenance to maintain cell homeostasis. When cells are stimulated by external pressure, HMGB1 can then translocate to the cytoplasm or migrate outside the cell membrane, participating in more complex regulation, including cell proliferation, autophagy, inflammation and immunity 13. Previous studies have shown that HMGB1 expression in tumour tissue is significantly higher than that in their normal counterparts and that HMGB1 levels correlate positively with tumour size, stage and prognosis of cancers, such as breast carcinoma 36, hepatocellular carcinoma 37, and lung cancer 17. Moreover, recent research has shown that extracellular HMGB1 triggers a series of functions by combining with different cell membrane receptor proteins (such as RAGE, TLR4, and CXCR4) 12, 16, while intracellular HMGB1 regulates autophagy over apoptosis 12. However, the intracellular release and localization of HMGB1 are still not completely understood in OSCC. We detected the interaction between EPHB4 and HMGB1 through mass spectrometry and co-IP experiments. Moreover, the CHX treatments showed that changes in EPHB4 expression affected the protein stability of HMGB1 in OSCC cell lines to a certain extent. The above results are consistent with the analysis of the positive correlation between EphB4 and HMGB1 by GEPIA (http://gepia.cancer-pku.cn/) data, which provides a more reliable basis for our follow-up research. Subsequent experiments showed that knocking down HMGB1 inhibited the proliferation, migration and invasion of OSCC cell lines without affecting EPHB4 expression, indicating that EPHB4 may regulate the biological functions of OSCC cells by targeting HMGB1.

The transcription factor NF-κB plays a wide role in inflammation and other natural or pathological processes 38-40. Activated NF-κB homo- or heterodimers translocate into the nucleus, alone or with other transcription factors, and activate downstream genes, thereby regulating survival, inflammation, the immune system, and the proliferation and metastasis of mammary carcinoma, which may be a useful strategy for inhibiting tumour malignancy 41-43. In addition, accumulating evidence has revealed that HMGB1 affects the malignant progression of tumours via NF-κB in different types of cancer. For instance, miR-449a was verified to suppress the proliferation and metastasis of tumours in lung cancer by targeting an HMGB1-mediated NF-κB signalling pathway 14. Furthermore, HMGB1 knockdown was reported to enhance tumour cell viability and arrest apoptosis-related proteins through NF-κB in HepG2 cells 15. In this study, we found that P-P65 expression decreased following HMGB1 knockdown. Intriguingly, we also found that the NF-κB pathway inhibitor JSH-23 could effectively reverse the proliferation and metastasis-promoting effects of EPHB4, which indicated that EPHB4 may promote the malignant function of OSCC cells through HMGB1-mediated activation of the NF-κB pathway.

In summary, our research demonstrated that EPHB4 was highly expressed in OSCC tissues, and its expression usually correlated with the clinical characteristics of OSCC patients. Then, we verified that EPHB4 promoted the growth and metastasis of OSCC in cell experiments and nude mouse xenograft models. Significantly, the mechanism of EPHB4 activity in OSCC was still not clear; thus, we further clarified that EPHB4 could activate the NF-κB pathway by interacting with HMGB1 to promote OSCC malignant progression. Exploration of the mechanism of EphB4 suggests that we may regulate the tumour microenvironment of OSCC through the HMGB1-mediated NF-κB signalling pathway. At present, we have performed a preliminary study on the function of EPHB4 in regulating the proliferation and metastasis of OSCC. In the future, we will continue to explore more roles of the EPHB4-HMGB1/NF-κB pathway in the OSCC tumour microenvironment, such as the regulation of tumour immunity. Based on the above research, our study may provide a relatively novel perspective to explore the role of EPHB4 in tumours and uncover the potential of EPHB4 as a target for OSCC molecular therapy.

## Supplementary Material

Supplementary figures.

## Figures and Tables

**Table A:** siRNA sequences for the EPHB4 and HMGB1 gene

Gene		5'-3' sequences
EPHB4	S1	GACGGACAGTTCACAGTCA
	S2	CCCAGCCAATAGCCACTCTAA
HMGB1	S1	GGAGAUCCUAAGAAGCCGATT
	S2	GGGAGGAGCAUAA GAAGAATT
SiNC		UUCUCCGAACGUGUCACGUdTdT

**Table B:** Primer sequences for EPHB4 and HMGB1

Gene	Forward	Reverse
EPHB4	CGCACCTACGAAGTGTGTGA	GTCCGCATCGCTCTCATAGTA
HMGB1	GTGCAAACTTGTCGGGAGGA	TCTCTTTCATAACGGGCCTT
GAPDH	GAGTCAACGGATTTGGTCGT	GACAAGCTTCCCGTTCTCAG

**Table 1:** EPHB4 Expression in OSCC and ANC Tissues

	Cases	High expression	Low expression	*P* value
OSCC	65	33 (50.77%)	32 (49.23%)	
ANC	65	20 (30.77%)	45 (69.23%)	0.01

**Table 2:** Correlation of EPHB4 Expression and OSCC Characteristics

	Cases	EPHB4 Expression		*P* value
		High (N=33)	Low (N=32)	
**Age**				
≤40	26	15 (57.69%)	11 (42.31%)	
>40	39	18 (46.15%)	21 (53.85%)	0.370
**Gender**				
Male	38	18 (47.37%)	20 (52.63%)	
Female	27	15 (55.56%)	12 (44.44%)	0.523
**Differentiation**				
High	27	7 (25.93%)	20 (74.07%)	
Medium	22	15 (68.18%)	7 (31.82%)	
Low	16	11 (68.75%)	5 (31.25%)	0.002
**Lymph node metastasis**				
No	32	11 (34.38%)	21 (65.62%)	
Yes	33	22 (66.67%)	11 (33.33%)	0.009
**Clinical stage**				
1	17	6 (35.29%)	11 (64.71%)	
2	22	10 (45.45%)	12 (54.55%)	
3	16	10 (62.50%)	6 (37.50%)	
4	10	7 (70.00%)	3 (30.00%)	0.043

**Table 3:** 22 identified proteins that bind to EPHB4 in OSCC cells

Gene Symbol	Coverage [%]	Gene Symbol	Coverage [%]
EPHB4	53	DBNL	3
KRT6A	27	CALR	2
HMGB1	13	CSDE1	2
RPL15	12	NR1H4	2
EIF5A	11	RBM39	2
S100A6	10	SLC3A2	2
ACTN4	9	TFRC	2
CRYAB	7	ADCY2	1
RPL14	6	ITGB4	1
CANX	4	MBTD1	1
ECH1	4	TRAF4	1

## Abbreviations

OSCC: Oral squamous cell carcinoma
EPH: erythropoietin-producing human hepatocellular
RTKs: receptor tyrosine kinases
