# EPHB4 Regulates the Proliferation and Metastasis of Oral Squamous Cell Carcinoma through the HMGB1/NF-κB Signalling Pathway: Erratum

**DOI:** 10.7150/jca.87278

**Published:** 2023-08-06

**Authors:** Chen Yi, Xiliu Zhang, Hongyu Li, Guanhui Chen, Binghui Zeng, Yiming Li, Chao Wang, Yi He, Xun Chen, Zixian Huang, Dongsheng Yu

**Affiliations:** 1 Department of Oral and Maxillofacial Surgery, Hospital of Stomatology, Guanghua School of Stomatology, Sun Yat-sen University. Guangzhou, Guangdong, China, 510055.; 2 Guangdong Provincial Key Laboratory of Stomatology. Guangzhou, Guangdong, China, 510055.; 3 Department of Stomatology, the Seventh Affiliated Hospital, Sun Yat-sen University, Shenzhen. Guangdong, China, 518107.; 4 Department of Oral and Maxillofacial Surgery, Sun Yat-sen Memorial Hospital, Sun Yat-sen University, Guangzhou, Guangdong, China, 510120.

In the original version of our article, there was an error in Fig. 3E. Specifically, the bar chart image of SCC9 Invasion in Figure 3E is incorrect. The correct image is provided below. This correction will not affect the results and conclusions. The authors apologize for any inconvenience this may have caused.

## Figures and Tables

**Figure 3:**
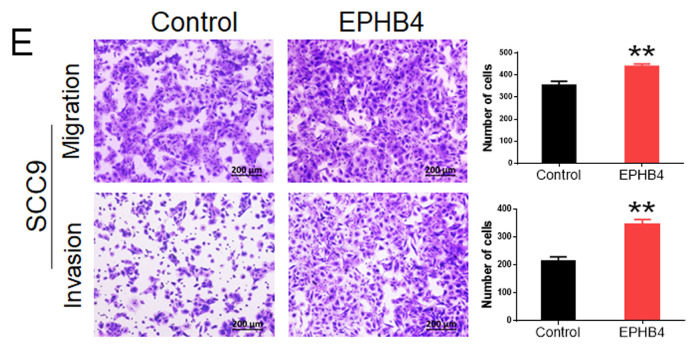
E. Transwell assays demonstrated that the overexpression of EPHB4 promoted the migration and invasion ability of SCC9 and UM1 cells. (scale bar, 200 μm)

