# Supplementary material for: EPHB4 Regulates the Proliferation and Metastasis of Oral Squamous Cell Carcinoma through the HMGB1/NF-κB Signalling Pathway
Source: J Cancer. 2021 Aug 20;12(20):5999–6011. doi: 10.7150/jca.59331 (PMC8425198; doi:10.7150/jca.59331)
Supplement: Supplementary file 1 — Supplementary figures. [file jcav12p5999s1.pdf]

Supplemental data

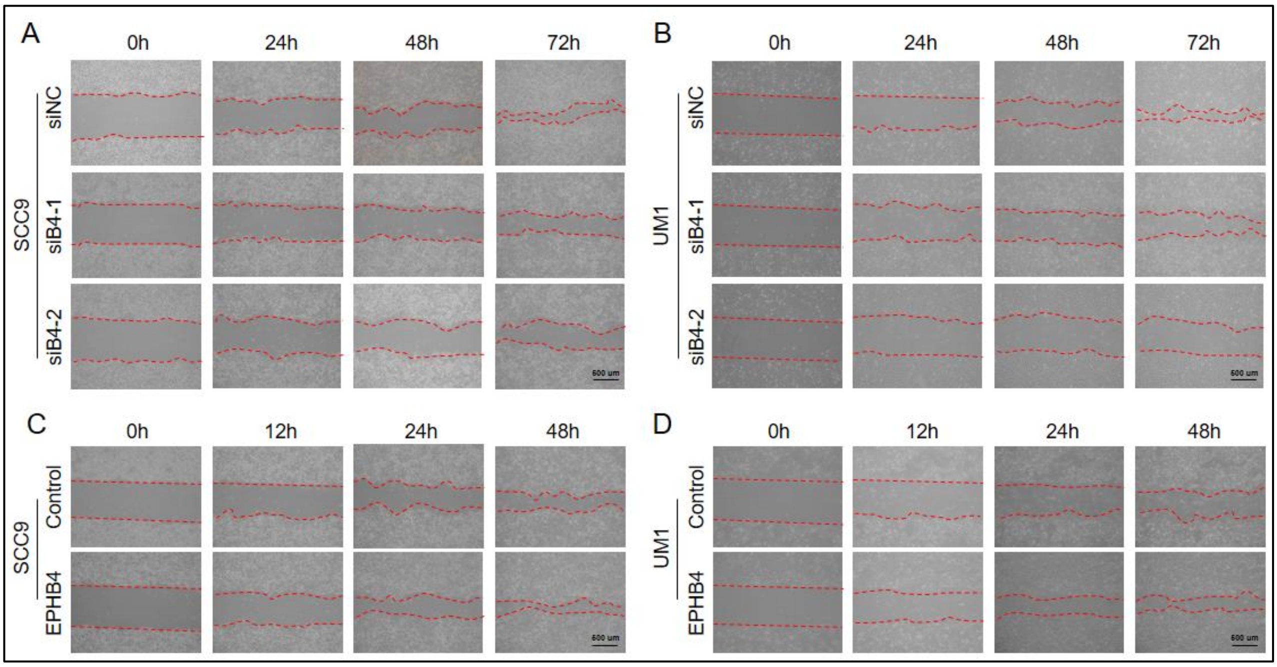

Figure 1s

A-B. Wound healing assays showed that the downregulation of EPHB4 inhibited the migration abilities of SCC9 and UM1 cells.

C-D. Wound healing assays showed that the up-regulation of EPHB4 promoted the migration abilities of SCC9 and UM1 cells.

(scale bar size: 500 μm).

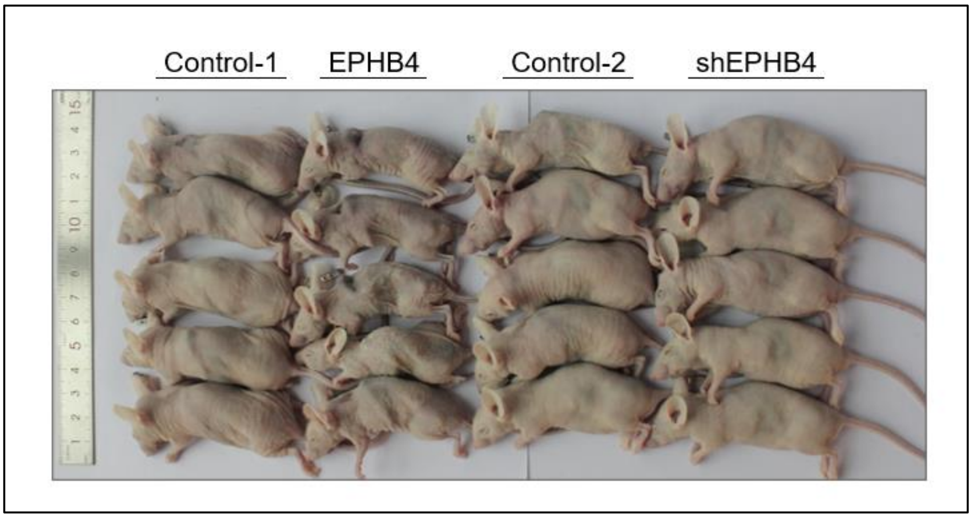

Figure 2s

Tongue xenografts mice models before taking specimens.

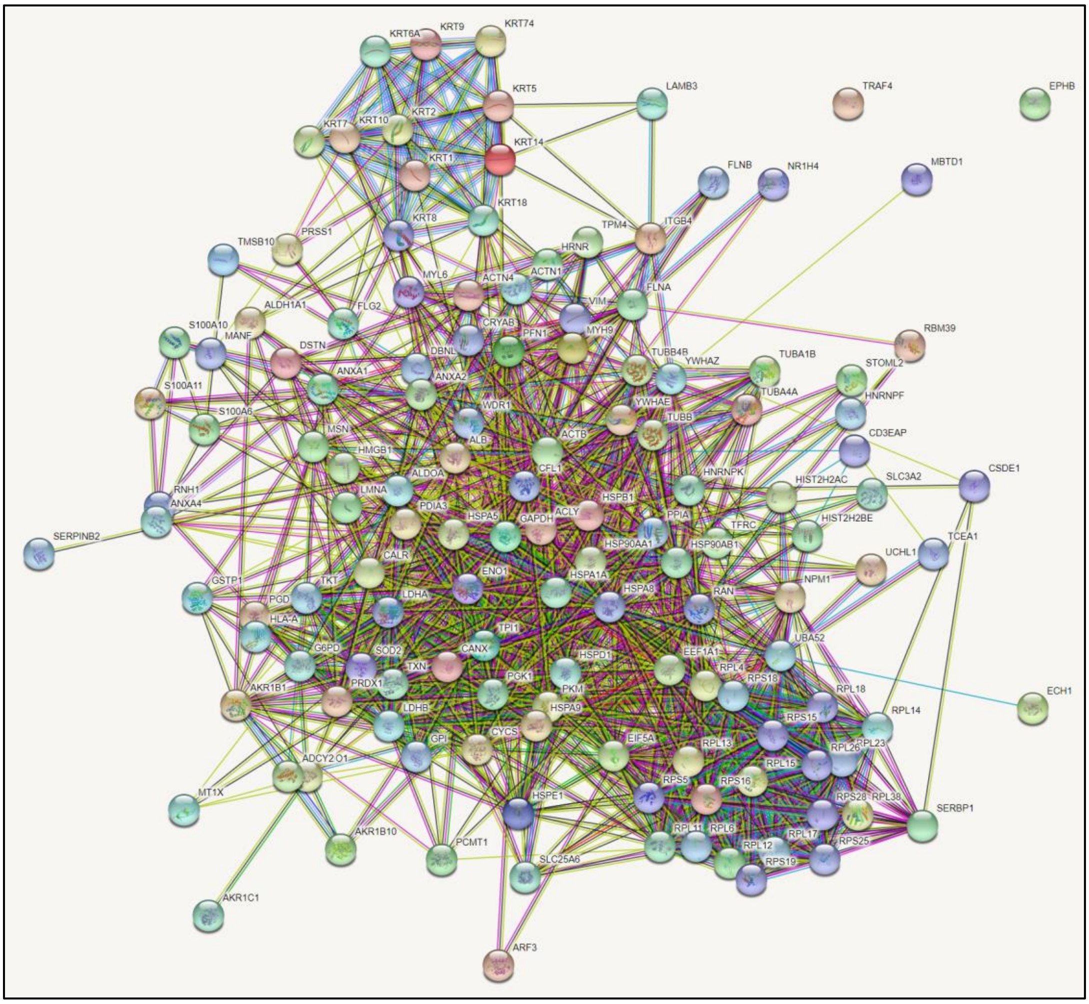

Figure 3s

Mass spectrometry experiments detected 137 proteins that bind to EPHB4 in SCC9 cells. Proteins that interact with the internal control IgG were not excluded.

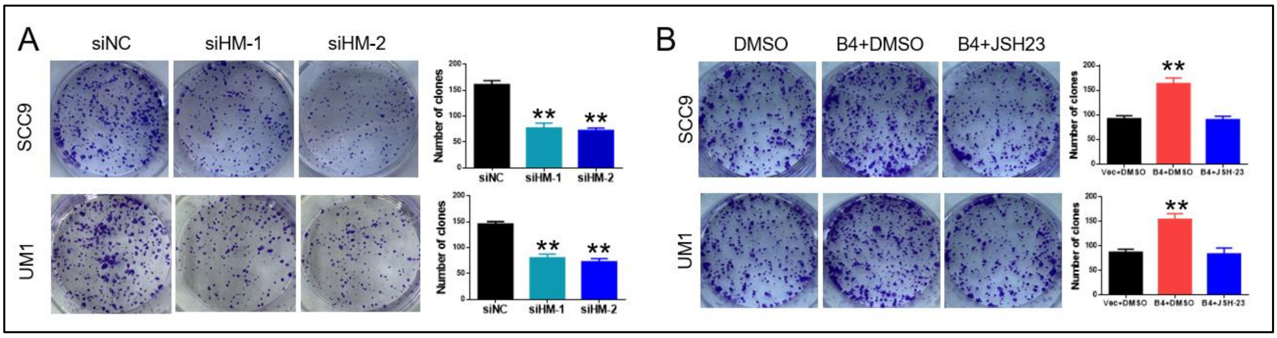

Figure 4s

A. After inhibiting HMGB1, the clone formation ability of OSCC cells decreased;

B. After using the NF-κB inhibitor JSH-23, the clone formation ability of EPHB4 overexpressing cells decreased compared to the control group;
